# Inflammatory biomarkers in depression: scoping review

**DOI:** 10.1192/bjo.2024.787

**Published:** 2024-09-30

**Authors:** Walter Paganin, Sabrina Signorini

**Affiliations:** School of Neuroscience, University of Tor Vergata, Italy; Studio Psicologia Signorini, Italy

**Keywords:** Depression, biomarkers, pro-inflammatory cytokines, neuroinflammation, review

## Abstract

**Background:**

Inflammation is increasingly recognised as a fundamental component of the pathophysiology of major depressive disorder (MDD), with a variety of inflammatory biomarkers playing pivotal roles. These markers are closely linked to both the severity of symptoms and the responsiveness to treatments in MDD.

**Aims:**

This scoping review aims to explore the scientific literature investigating the complex relationships between inflammatory biomarkers and depression, by identifying new studies and critical issues in current research.

**Method:**

Following the PRISMA Extension for Scoping Reviews guidelines, we systematically searched databases including PubMed, Scopus, PsycINFO, Open Grey and Cochrane Library. Our search focused on articles published from 1 January 2020 to 1 May 2024. We included studies evaluating inflammatory biomarkers in adult patients with MDD, utilising observational and randomised controlled trial designs, and review studies.

**Results:**

Our analysis examined 44 studies on the complex interplay between inflammation and its multiple effects on MDD. Significant associations between specific inflammatory biomarkers and depression severity were found, requiring cautious interpretation. We also highlight several methodological limitations in the current studies, which warrant caution in directly applying these findings to clinical practice. However, identified methodologies show potential for using these biomarkers as diagnostic tools or therapeutic targets, including anti-inflammatory interventions.

**Conclusions:**

The findings emphasise the need for sophisticated, integrative research to understand inflammation's role in MDD. Future studies should identify specific biomarker panels for diagnosing depression and bridging peripheral biomarker measurements with central neuroinflammatory processes, leading to better diagnostic and treatment strategies.

Major depressive disorder (MDD) is one of the most widespread mental disorders globally and a major cause of disability, affecting personal, family, professional and social aspects of patients’ lives. According to the Institute for Health Metrics and Evaluation, approximately 280 million people had depression in 2020,^1^ and the incidence of MDD increased by 28% following the COVID-19 pandemic, particularly among women and young people.^2^ Recent research has emphasised the role of biological markers in indicating the presence and severity of MDD. These markers potentially include genetic, neuroendocrine and inflammatory indicators, which are crucial for diagnosis, guiding therapy and the development of new drugs.^3–7^ Over 9000 studies have investigated the immune system's role in depression, and particularly how inflammation contributes to MDD.^8^ The relationship was initially linked to depressive symptoms and increased cortisol levels resulting from disruptions in the hypothalamic-pituitary-adrenal and hypothalamic-pituitary-gonadal axes.^9,10^ However, the aetiology of depression is complex, involving genetic, traumatic, environmental and psychosocial factors, which complicates the identification of specific biomarkers. Current studies are examining various biomarkers, such as cytokines and neuropeptides, focusing on neuroinflammation and elements of both the innate and adaptive immune systems.^8,11,12^ Researchers are also evaluating potential clinical immunotherapies for treating depression, rethinking the pathophysiological mechanisms primarily from an immunological perspective. The immune system is divided into two main categories: the innate and adaptive immune systems. The innate system acts as the first line of defence against antigens, primarily using macrophages to detect and destroy pathogens and produce cytokines essential for the inflammatory response. In contrast, the adaptive system, which includes T cells and B cells, mounts a more targeted and delayed response using cytokines and antibodies once specific antigens are recognised.^13^ It is known that inflammatory signaling at both peripheral and central nervous system levels initiates and amplifies inflammatory processes.^14^ Inflammatory signalling at both the peripheral and central nervous system levels initiates and amplifies inflammatory processes. It begins through the role of intracellular transcription factors, such as nuclear factor kappa B (NF-κB) and activator protein 1 (AP-1), which promote the expression and production of pro-inflammatory cytokines like tumor necrosis factor-alpha (TNF-α), interleukin-6 (IL-6) and interleukin-1β (IL-1β).^12^ These cytokines increase the activity of enzymes called cyclooxygenases (COX-1 and COX-2), which subsequently raise levels of prostaglandins and intensify the inflammatory response. In the central nervous system, microglia transform into an amoeboid shape releasing pro-inflammatory cytokines, significantly affecting neurotransmitter systems and activating the mitogen-activated protein kinase (MAPK). This activation enhances the activity of presynaptic reuptake pumps for serotonin (5-HT), dopamine and norepinephrine, reducing their availability across synapses. Moreover, cytokines activate the enzyme indoleamine 2,3-dioxygenase, which converts tryptophan to kynurenine, reducing tryptophan availability for serotonin synthesis. Excess kynurenine is further metabolised by activated microglia into quinolinic acid, which stimulates the N-methyl-D-aspartate receptor (NMDAR) and promotes the release of glutamate. Pro-inflammatory cytokines lead to a decrease in glutamate reuptake and an increase in its release, which, when linked to extrasynaptic NMDAR receptors, reduces the synthesis of brain-derived neurotrophic factor (BDNF). BDNF is crucial for neuronal integrity and neurogenesis.^12^ Cytokines, small peptides essential for cellular communication within the immune system, include interleukins, interferons, chemokines, lymphokines and the tumor necrosis factor superfamily.^15,16^ Pro-inflammatory cytokines, such as IL-1β, IL-6, TNF-α and IL-8, are critical for initiating the immune response to infections, injuries or stress, causing typical signs of inflammation such as redness, swelling, heat and pain. In contrast, anti-inflammatory cytokines such as IL-10 and transforming growth factor-beta (TGF-β) help modulate and reduce inflammation, facilitating tissue repair and preventing excessive tissue damage. Maintaining a balance between these cytokines is critical, as an excess of pro-inflammatory cytokines can lead to chronic inflammatory diseases such as rheumatoid arthritis or autoimmune conditions, whereas a deficiency can increase susceptibility to infections.^17^ Cytokines are vital in the immune response and also influence the nervous system by inducing neuroinflammation.^18^ Some of these, studied in relation to depression, appear to play a significant role (see Table 1, adapted from Sakamoto et al).^19^

**Table 1:** Cytokines and other inflammatory biomarkers associated with depression

Molecule	Main function in neuroinflammatory processes	Detection location^a^	Values detected in depression^19^
IL-1β	Promotes inflammation, induces fever and activates immune responses	Blood, CSF, brain tissue	Increased
IL-2	Stimulates growth and differentiation of T cells, regulates immune responses	Blood, CSF	Increased
IL-3	Supports growth and differentiation of haematopoietic cells, involved in neuroinflammation	Blood, CSF	
IL-4	Differentiation of Th2 cells, anti-inflammatory properties	Blood, CSF	Decreased
IL-5	Growth and activation of eosinophils, involved in immune responses	Blood, CSF	
IL-6	Promotes inflammation, stimulates acute phase responses and affects brain function	Blood, CSF, brain tissue	Increased
IL-7	Development and survival of T cells, involved in immune homeostasis	Blood	
IL-8	Chemotaxis of neutrophils, promotes inflammation	Blood, CSF	Increased
IL-9	Supports growth of T cells and mast cells, involved in allergic responses	Blood	
IL-10	Anti-inflammatory cytokine, regulates immune responses	Blood, CSF	
IL-11	Anti-inflammatory effects, stimulates haematopoiesis	Blood, CSF	
IL-12	Differentiation of Th1 cells, enhances cytotoxic activity	Blood, CSF	Increased
IL-13	Anti-inflammatory, involved in allergic responses	Blood, CSF	
IL-17A	Promotes inflammation, recruits neutrophils	Blood, CSF	
IL-18	Induces IFN-γ production, activates immune cells	Blood, CSF	
IFN-α	Antiviral activity, modulates immune responses, affects brain function	Blood, CSF	Increased
CCL2 (MCP-1)	Chemotaxis of monocytes, promotes inflammation	Blood, CSF	Increased
CCL3 (MIP-1α)	Chemotaxis of monocytes and T cells, activates immune cells	Blood, CSF	
CCL4 (MIP-1β)	Chemotaxis of macrophages, T cells, and dendritic cells	Blood, CSF	Decreased
CCL5 (RANTES)	Chemotaxis of T cells, eosinophils, and basophils, activates immune responses	Blood, CSF	
CCL11 (Eotaxin-1)	Recruits eosinophils, involved in allergic inflammation	Blood	
IFN-γ	Activates macrophages, promotes Th1 responses	Blood, CSF	
TNF	Promotes inflammation, induces apoptosis	Blood, CSF, brain tissue	Increased
TGF	Regulates immune responses, promotes tissue regeneration	Blood, CSF, brain tissue	Decreased
BDNF	Promotes neuron survival, growth and differentiation	Blood, CSF, brain tissue	Decreased
CRP	Marker of systemic inflammation, acute phase response	Blood	Increased
CXCL4	Chemotaxis of immune cells, modulates inflammation	Blood, platelets	Increased
CXCL7	Chemotaxis of neutrophils, promotes inflammation	Blood, platelets	Increased
VEGF	Promotes angiogenesis, vascular permeability	Blood, CSF	Increased or stable
IBA1	Microglial activation marker, involved in neuroinflammation	Brain tissue	Increased
TLR3	Recognises viral dsRNA, activates innate immune response	Blood, brain tissue	Increased
TLR4	Recognises bacterial LPS, activates innate immune response	Blood, brain tissue	Increased

IL, interleukin; CSF, cerebrospinal fluid; Th, T helper cells; IFN, interferon; CCL, C-C motif chemokine ligand; MCP, monocyte chemoattractant protein; MIP, macrophage inflammatory protein; RANTES, regulated upon activation, normal T cell expressed and secreted; TNF, tumor necrosis factor; TGF, transforming growth factor; BDNF, brain-derived neurotrophic factor; CRP, C-reactive protein; CXCL, C-X-C motif chemokine ligand; VEGF, vascular endothelial growth factor; IBA1, ionized calcium-binding adapter molecule 1; TLR, toll-like receptor; dsRNA, double-stranded ribonucleic acid; LPS, lipopolysaccharide.

a. Blood refers to serum or plasma samples. Brain tissue refers to tissue samples collected during autopsy or research studies.

## Research implications

Researchers are studying how cytokine levels during neuroinflammation might influence the effectiveness of antidepressant treatments.^20^ This connection underscores the importance of understanding cytokines not only for immune regulation, but also for their potential impact on mental health. Research has identified numerous inflammatory markers, ranging from well-studied to lesser known, that could play a role in depressive disorders.^21,22^ The complex interplay between the immune system and psychiatric conditions has spurred research into the need for regulated anti-inflammatory responses to alleviate depressive symptoms.^23,24^ This interest also extends to certain genetic biomarkers that indicate a predisposition to MDD, resulting from genetic mutations that may also influence its severity.^25^ Studies have shown how personal attachment styles and relational dynamics modulate the functioning of the immune system and can alter levels of inflammation.^26,27^ Further research has explored these interactions, confirming how specific factors such as social relationships, including social isolation, loneliness, and lack of social support, can influence the immune system.^28^ Attention to pro-inflammatory cytokines has led to their consideration in screening and predicting responses to antidepressant treatment.^12^ Evidence indicates that individuals with depression who do not respond to monoamine reuptake inhibitors exhibit abnormal levels of various pro-inflammatory activity markers compared with those who respond to pharmacotherapy. This link has led to the hypothesis that, in a subpopulation of individuals with depression, the inflammatory component might be central to the depressive condition.^29^ For example, elevated levels of circulating inflammatory markers predict a poor response to selective serotonin reuptake inhibitors, whereas high levels of these markers predict an enhanced response to tricyclic antidepressants, ketamine and electroconvulsive therapy.^20,30,31^ In neuroinflammation, increased activity of cyclooxygenase (COX-1 and COX-2) leads to higher levels of prostaglandins, which further promote inflammation.^32^ Therefore, inhibiting the enzymatic activity of COX-1 and COX-2 could be a target for non-steroidal anti-inflammatory drugs, proposed as new neuroimmunological treatments for cases of depression that are difficult to treat.^22^ Emerging evidence from recent studies highlights the potential of anti-inflammatory treatments across different age groups with depression. A study in adults revealed that white blood cell counts, a broad marker of the immune system, do not predict treatment responses in bipolar disorder, indicating the complexity of immune interactions in psychiatric conditions.^33^ Meanwhile, a meta-analysis of anti-inflammatory treatments in children and adolescents with depressive symptoms found a small but significant effect, particularly from omega-3 fatty acids, in reducing depressive symptoms. This suggests a key role for inflammatory pathways in the supplementary treatment of young individuals with depressive disorders.^34^ The importance of considering age as a critical variable in the diagnosis and treatment protocols for depression is emphasised. A well-known phenomenon in individuals over 50 years of age is inflammaging, characterised by chronic low-level inflammation and an increase in inflammatory cytokines during the ageing process.^35^ This condition can have negative health consequences, as chronic inflammation in those aged over 50 years reflects an age-related alteration in the immune system, leading to an exaggerated and dysfunctional inflammatory response. Understanding inflammaging is essential for developing therapeutic strategies aimed at modulating pro-inflammatory cytokines to promote health in ageing. This also suggests the need for further longitudinal studies to explore these dynamics more thoroughly. Research consistently shows that individuals with depressive disorders exhibit higher levels of various cytokines compared with controls, with notable increases in C-reactive protein (CRP), IL-6, IL-1β and TNF-α.^36–38^ Although substantial progress has been made in understanding the immune system dysregulation in depression, many questions remain unanswered. Clinical research has yielded conflicting results regarding the efficacy of anti-inflammatory agents in treating depression.^39^ Like other neuropsychiatric disorders, depression is not a singular disease, but a heterogeneous syndrome, characterised by a variety of symptoms and diverse responses to treatments.^40^ Although increased inflammatory cytokines are observed in some patients with depression, this does not necessarily indicate that inflammation is a universal aspect of the disorder. The levels of these cytokines vary among individuals with depression, and not all individuals with elevated cytokine levels experience depression. Furthermore, factors such as body mass index and blood pressure may also affect these markers, yet their impact on the link between depression and inflammation has not been thoroughly investigated in most studies. The reasons for these variations – whether they stem from an incomplete understanding of immune system anomalies, comorbidities or a focus on specific cytokines that enhance mood – remain elusive, as does the influence of environmental factors on inflammation.^39^

The strong correlation between inflammatory processes and MDD is increasingly emphasised in the scientific literature. However, recent studies urge caution due to the complex relationship between cytokine levels in peripheral blood, cerebrospinal fluid (CSF) and nervous tissue. Therefore, we conducted a scoping review on inflammatory biomarkers in depression, which is essential not only for framing the current context but also for identifying potential gaps in the existing literature. This review systematically summarises the knowledge acquired, examining both the coherence and discrepancies between recent studies and critically evaluating their implications for clinical practice by integrating the most recent data with pre-existing evidence. This scoping review represents a crucial opportunity to synthesise a large body of research, presenting the state of the art to guide future scientific efforts in immunopsychiatry, and proposing new study hypotheses and directions for further treatments.

## Method

### Conceptual framework and objectives

This scoping review was conducted in accordance with the PRISMA Extension for Scoping Reviews (PRISMA-ScR) guidelines,^41^ with the aim of mapping the literature related to inflammatory biomarkers in depression, exploring the diversity of available studies and identifying key research areas. The objective was to provide a comprehensive understanding of the correlations between inflammation and depression through a variety of evidence sources and study contexts.

### Search strategy

Focusing on research from the past 5 years, our objective has been to identify and assess emerging trends in the study of inflammatory biomarkers in depression, a field characterised by rapid development and significant advancements. This temporal limitation enabled us to focus on the most recent studies that address current scientific challenges, thereby ensuring a synthesis that is up-to-date with the scientific literature. The search strategy, tailored to meet the goals of this scoping review, facilitated the inclusion of a broad spectrum of research, encompassing literature reviews, randomised controlled trials (RCTs), non-RCTs and observational studies. In PubMed, our search yielded 417 relevant articles published between 1 January 2020 and 1 May 2024, using established methodologies with the search string: ‘depressive disorders AND inflammatory markers NOT covid’ across ‘All fields’. In SCOPUS, for the same period, the bibliographic search was broadened using the string in ‘TITLE-ABS-KEY’: (markers of depression-inflammation correlation) AND PUBYEAR > 2019 AND PUBYEAR < 2025 AND (LIMIT-TO (DOCTYPE,‘ar’)) AND (EXCLUDE (SUBJAREA,‘AGRI’) OR EXCLUDE (SUBJAREA,‘CENG’) OR EXCLUDE (SUBJAREA,‘EART’) OR EXCLUDE (SUBJAREA,‘COMP’) OR EXCLUDE (SUBJAREA,‘CHEM’) OR EXCLUDE (SUBJAREA,‘ENVI’) OR EXCLUDE (SUBJAREA,‘SOCI’) OR EXCLUDE (SUBJAREA,‘MULT’)) AND (LIMIT-TO (LANGUAGE,‘English’)) AND (LIMIT-TO (EXACTKEYWORD,‘Human’)), resulting in 230 records. The PsycINFO database, using the same search string and time frame as PubMed, resulted in 259 records. Conversely, in Open Grey, with the string: ‘Biomarkers of inflammation in depressive disorders’ applied within the same period, no records were found. Finally, in the Cochrane Library, a search from 1 January 2020 to 1 May 2024, with the string: ‘depressive disorders AND inflammatory markers NOT covid’ performed in the ‘Title, Abstract, Keyword’ section of the database without other restrictions, produced two reviews.

### Study selection

During the study selection phase, two independent reviewers (W.P. and S.S.) anonymously screened the titles and abstracts of the retrieved articles. To ensure the impartiality of the review process and minimise the risk of bias, the identity of the study authors, their institutional affiliations and details about the study outcomes were concealed during the initial screening phase. Discrepancies between reviewers were resolved through discussion and consensus, or, if necessary, by consulting a third expert reviewer. Potentially relevant reports were then fully assessed based on explicit selection criteria. Inclusion criteria were (a) human studies, (b) investigations on patients with depression and controls, (c) publication between 2020 and 2024, and (d) assessment of inflammatory biomarkers in relation to depression. Exclusion criteria included (a) non-human context, (b) reporting duplication, (c) studies where quantitative assessments of inflammation markers had not been performed, (d) studies concerning SARS-CoV-2 disease as a confounding factor and (e) studies on paediatric and geriatric populations.

### Data extraction and quality assessment

Data extraction and the assessment of the quality of the included studies were conducted by two blinded reviewers (W.P. and S.S.), using predefined electronic forms, ensuring consistency and accuracy across the review process. The reviewers independently collected specific information, such as authors, publication year, study context and main findings, according to a standardised template. This aimed to systematically capture the essential aspects of each study regarding inflammatory biomarkers in depression, facilitating the identification of common themes, trends and existing research gaps. Simultaneously, the quality of each study was evaluated to ensure the reliability of the findings, with any discrepancies between reviewers resolved through discussion and consensus. This combined approach aligns with the scoping review's goals, focusing on comprehensively mapping out the field without conducting a formal quality evaluation of individual studies.

**Fig. 1:**
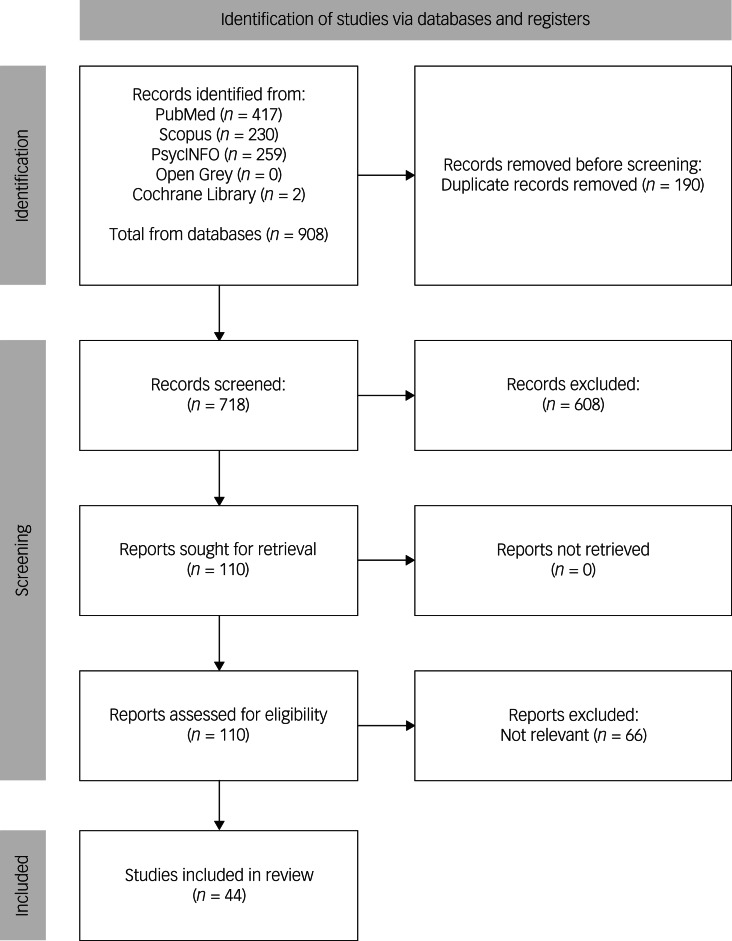
PRISMA flow diagram of the study selection process.

Our search yielded a total of 908 potentially relevant records. Of these, 190 were excluded as duplicates, and 608 were excluded based on their titles or abstracts as they were not relevant. The remaining 110 studies underwent a more detailed assessment, resulting in the exclusion of 66 articles because they did not meet the inclusion criteria. Ultimately, 44 studies met the inclusion criteria (see Fig. 1). This search was conducted up to 1 May 2024. All articles that met the inclusion criteria are reviewed here. Applying the PRISMA-ScR guidelines enhanced the transparency and systematic nature of our scoping review, enabling us to effectively explore the field of study.

## Results

Our comprehensive analysis of 44 studies, including 30 observational studies, 2 RCTs and 12 reviews, confirmed a clear association between elevated levels of inflammatory biomarkers and the presence of depressive symptoms across various subtypes of major depression (melancholic, atypical, anxious) and different states of remission.^42–48^ For a complete summary of the results, see Tables 2–4. Details of these studies are given below.

**Table 2:** Review studies

Article	Methodology	Results
Nobis et al^49^	Scientific review of the literature exploring biological markers for major depression. The review includes references to clinical trials, cohort studies, cross-sectional studies and systematic reviews that have examined biological markers associated with depression	This article synthesised existing research on various peripheral biomarkers for depression, highlighting findings on inflammatory markers, neurotrophic factors and others that may contribute to the pathophysiology of MDD. As a review, it does not present original research, but depends on the selection and interpretation of existing studies, which may introduce selection bias or highlight inconsistent findings across studies
Ye et al^50^	A large-scale meta-analysis randomised study conducted using data from the UK Biobank to assess the causality between inflammation, as indicated by CRP levels, and the onset of depression over the past 2 weeks. Depression was measured using the Patient Health Questionnaire (PHQ)-9 in a sample of 144 890 individuals	Magnetic resonance imaging analyses suggested that higher genetically predicted IL-6 activity was associated with an increased risk of depressive symptoms, whereas a higher genetically predicted concentration of CRP was associated with a decreased risk of depressive and anxious symptoms
Carniel and da Rocha^43^	Systematic review and meta-analysis on inflammatory biomarkers for the management of depression	In patients with mood disorders, the importance of BDNF is assessed in the management of depression and inflammatory markers (IL-6, IL-1β, TNF-α, CRP) indicate a potential state of depression
Mac Giollabhui et al^51^	Application of meta-analysis and meta-regression to study the behaviour of three inflammatory biomarkers	It found significant associations between elevated levels of CRP and IL-6 and the likelihood of depressive symptoms. No significant associations were observed for TNF-α, indicating that not all inflammatory markers might be equally indicative of depression. This nuanced understanding could help refine the targeting of inflammatory pathways in the treatment and diagnosis of depression
Debnath et al^52^	Narrative review on state-of-the-art analysis on assessing depression severity (using the HRSD scale), identifying inflammatory markers, and examining results through neuroimaging	Elevated levels of inflammatory markers (IL-1β, TNF-α and IL-6) were found in patients with depression, both in peripheral blood and cerebrospinal fluid. Post-mortem brain studies revealed significant levels of other IRS cytokines, including IL-10, TNF-α, IL-1A and IL-8
Kopra et al^53^	Selection of 62 articles, of which 31 were included in the qualitative analysis. Out of these 31 studies, nine were human-based and 22 were on rodents	Ketamine appears to induce anti-inflammatory effects in at least a subset of patients with depression. Suggestions for future research include investigating markers in the central nervous system and examining the clinical relevance of inflammatory changes
Harsanyi et al^54^	This systematic review compiles diverse types of studies, such as observational, meta-analytic and literature reviews, focusing on varied populations. It encompasses methodologies ranging from cytokine analysis and monocyte profiling to multi-assay biological tests and mindfulness-based interventions. Specifically, the review details studies on cytokine gene expression and the impact of mindfulness interventions on biomarkers and low-grade inflammation	The document identifies correlations between cytokine levels and depression, alterations in monocyte profiles and increased cytokine expression in suicide victims, suggesting potential drug targets for depression. It also reports that mindfulness-based interventions effectively reduce depressive symptoms and inflammatory markers. However, methodological challenges such as varied mindfulness protocols, inconsistent sample sizes and potential biases exist, with effectiveness possibly influenced by participants’ psychological or motivational traits
Breit et al^55^	Scientific systematic review on inflammatory biomarkers	The study confirms that MDD is often linked to an overproduction of pro-inflammatory cytokines, representing a pro-inflammatory condition in some patients, characterised by severe clinical progression and treatment resistance. Peripheral inflammation may activate microglia, triggering a cascade that disrupts neurotransmitter balance. Pro-inflammatory markers may also affect the microstructure and integrity of white matter, with cytokine levels potentially predicting antidepressant treatment outcomes. Specifically, IL-6 may predict responses to ECT and antidepressant therapies
Reus et al^56^	Systematic review and meta-analysis examining recent advances in the role of inflammation in the pathophysiology of MDD and discussing possible new neurotherapeutic targets that may have antidepressant effects by modulating inflammation	The study summarises evidence supporting the role of both peripheral and cerebral inflammation in the pathogenesis of MDD. It highlights how anti-inflammatory drugs and nutritional strategies that reduce inflammation could potentially alleviate depressive symptoms. As a review, it notes limitations related to the variety and quality of the studies included
Gigase et al^57^	Systematic literature review and meta-analysis	The review focused on original research articles from peer-reviewed journals in English, investigating the correlation between cytokines or chemokines in plasma/serum and CSF. Studies with post-mortem samples, lacking baseline drug testing or with fewer than ten participants were excluded. Of the 29 studies considered, 21 were analysed, with eight excluded because of missing correlation coefficients. The findings revealed discrepancies between cytokine levels in blood and CSF, suggesting that blood cytokines might not accurately reflect central nervous system inflammation, affecting the diagnosis and treatment of psychiatric and neurological disorders
Chang et al^58^	Review of the scientific literature on the association between inflammation and depression, with a particular focus on the role of pro-inflammatory cytokines as biomarkers in the diagnosis and treatment of depression	Inflammation and increased pro-inflammatory cytokines are considered key pathogenetic factors in depression and high levels could be potential markers of disease severity. High levels of inflammatory cytokines are associated with treatment-resistant depression, suggesting that the presence of levels that may require anti-inflammatory therapy as a promising emerging strategy for treating depression
Varghese et al^59^	Review that synthesises information from various studies on heart disease and depression, without involving a specific study population. It employs a methodology of collecting, analysing and synthesising existing research to update the interactions of inflammatory biomarkers in patients with heart disease and depressive symptoms. The review aims to explore the potential of these biomarkers as predictive factors for both conditions, allowing the authors to evaluate the current understanding without generating new empirical data	The review emphasises the pathophysiological mechanisms connecting heart disease and depressive disorders, including endothelial dysfunction, hypothalamic-pituitary-adrenal axis dysregulation and abnormal platelet activity. It also discusses the specific roles of inflammatory biomarkers within this context. Although offering a substantial update on current research, the review underscores the need for more studies on this topic

MDD, major depressive disorder; CRP, C-reactive protein; IL, interleukin; BDNF, brain-derived neurotrophic factor; TNF, tumor necrosis factor; HRSD, Hamilton Rating Scale for Depression; IRS, inflammatory response system; ECT, electroconvulsive therapy; CSF, cerebrospinal fluid.

**Table 3:** Observational studies

Author	Type of study	Analysed sample	Results	Limitations
Felger et al^60^	Observational cross-sectional study	89 participants (males and females), aged 21-65 years, diagnosed with MDD or current depressive episode of bipolar disorder	The inflammatory biomarkers CRP in plasma (>3 mg/L) correlated with more severe depressive symptoms, especially anhedonia and reduced motivation, and were associated with increased levels of TNF and soluble IL-6 receptors in CSF	Cross-sectional study design limits the ability to determine causal relationships. Sample size and absence of healthy controls or patients with other psychiatric disorders, limiting the generalisability of the results
Brunoni et al^61^	Observational cross-sectional study	245 individuals with MDD and 59 individuals with bipolar depression. No specific information on the exact number of males and females or the mean age of the participants	The study results revealed significant variations in immune-inflammatory profiles between unipolar and bipolar depression. Cytokines studied in patients with MDD and bipolar depression include IL-1β, IL-6, TNF-α, IL-12, IL-18, CXCL-8, sTNFR1, sTNFR2, IL-10 and IL-33	Lack of a control group for comparison, and differences in medication exposure among participants
Pedraz-Petrozzi et al^42^	Observational case–control study	94 participants: 43 in the depression group and 51 in the healthy control group, with an approximate balance of men and women, aged between 18 and 65 years	Significant correlations were found between specific inflammatory markers and different aspects of fatigue in patients with depression and healthy controls. In the patient group, TNF-α was linked with overall depression severity (BDI-FS), cognitive fatigue and psychosocial fatigue. In healthy controls, IL-6 and TNF-α were associated with somatic fatigue, and IL-6 was also correlated with cognitive fatigue. These findings suggest a relationship between certain inflammatory markers and various fatigue dimensions across both groups	Limitations of the study could include the limited sample size, which could affect the generalisability of the findings. Additionally, the observational nature of the study may limit the ability to draw definitive conclusions about the causal relationship between depression, inflammation and fatigue
Milaneschi et al^62^	Observational cross-sectional study	A sample of patients with a current (*n* = 1100) or past (*n* = 753) diagnosis of MDD and healthy controls (*n* = 642)	Levels of tryptophan catabolites were found to correlate with specific clinical and biological features, including atypical symptoms and a pro-inflammatory state. Clinical studies are recommended to specifically focus on patients who exhibit clear evidence of dysregulation in the kynurenine pathway	Limitations include potential confounding factors, possibility of selection bias in the participant recruitment process and generalisability of the findings to other populations beyond the study sample
Gałecka et al^63^	Observational cross-sectional study that investigates the expression of certain inflammatory mediators in patients with MDDs compared with healthy controls	290 individuals, including 190 patients diagnosed with recurrent depressive disorders and 100 healthy volunteers. Patients were aged 20–67 years (mean age 47.51, s.d. = 11.18), including 117 women (61.6%) and 73 men (38.4%). Healthy volunteers: 65 women and 35 men, with a mean age of 41.29 years (s.d. = 13.50)	The study revealed that patients with MDD exhibited higher levels of pro-inflammatory cytokines (IL-17, IL-21 and IL-23) and lower levels of anti-inflammatory cytokines (IL-35) compared with healthy volunteers. Additionally, the study found that the expression of specific inflammatory genes (IL-17, IL-23 and IL-35) was significantly higher in patients with MDD compared with healthy controls	The study's cross-sectional design only identifies associations, not causality, between inflammatory processes and MDD. Measurements taken at a single time point may not capture dynamic changes, and reliance on self-reports and clinical scales can introduce variability in symptom reporting
Kappelmann et al^64^	Analytical observational study using existing data from different cohorts	Analyse three samples: MARS = 1058 patients STAR*D = 1143 patients UK Biobank = 110 010 individuals	Polygenic risk for CRP was found to be associated with changes in appetite and fatigue, whereas polygenic risk for TNF-α was associated with changes in appetite. Furthermore, high levels of CRP and TNF-α showed a strong association with depressive symptoms	Limitations of the study include variation in the structure of depressive symptoms between acutely ill patients and those in remission, as well as diversity in methods of measuring depressive symptoms among the different samples used in the study
Frank et al^65^	Observational randomised pooled analysis study of 15 population-based cohorts with a total of 56 351 participants, in both cross-sectional and longitudinal analyses	56 351 participants with individuals aged 18 years and over. Gender distribution balanced 48.5% men, 51.5% women	The study demonstrates a correlation between systemic inflammation and individual symptoms of depression. Higher levels of inflammatory markers (IL-6 and CRP) are associated with an increased likelihood of depressive symptoms	The observational design may introduce biases, and the lack of repeated measurements could affect the accuracy of inflammatory biomarker data. Additionally, it was not possible to account for the influence of anti-inflammatory drugs, antidepressants or anticoagulants, which could have altered the observed relationship between systemic inflammation and depressive symptoms
Poletti et al^66^	A cross-sectional observational study focusing on the comparative analysis of levels of various immuno-inflammatory biomarkers in patients with MDD and bipolar disorder, as well as in a healthy control group	208 patients hospitalised for major depressive episodes, comprising 127 with MDD and 81 with bipolar disorder, along with 32 healthy controls. Patients with major medical or neurological disorders, substance misuse or dependency, and inflammatory diseases were excluded. The plasma levels of 54 cytokines, chemokines and growth factors were analysed	The study found that an immune-inflammatory signature differentiates MDD and bipolar disorder with high accuracy (AUC = 97%). MDD was associated with elevated levels of both pro-inflammatory and regulatory markers (IL-1β, IL-6), whereas bipolar disorder was correlated with higher levels of specific inflammatory markers (CCL3, CCL4, CCL5, CCL11, IL-9, TNF-α)	Limited sample size and selection of patients from a single institution
Pitharouli et al^67^	A case–control study	26 894 patients with depression compared with 59 001 healthy individuals	CRP levels were significantly higher in patients with depression compared with the controls	Using a sample of individuals with complete data (approximately 86 000 people) rather than the entire UK Biobank database (500 000 people), which may affect the representativeness of depression cases in the sample
Lan et al^68^	Observational longitudinal study based on a specific sample of patients with MDD	54 drug-naïve patients aged 18-65 years, selected using the HRSD-17. All patients, previously drug-naïve, were treated with antidepressants (escitalopram or venlafaxine) for 4 consecutive weeks	The study identified significant associations between serological and epigenetic markers of inflammation and structural brain metrics in individuals with MDD. Higher levels of serum CRP were associated with reduced cortical thickness in several regions, including the left superior frontal gyrus, the left insula and the left superior temporal gyrus	Small sample size, which might limit the generalisability of the findings. The 4-week duration may not have been sufficient to observe the full therapeutic effects and changes in cytokine levels, which might need a longer time to stabilise. Measured cytokine levels in plasma, not CSF, which might not fully reflect central nervous system inflammation
Palmos et al^69^	Cross-sectional study focusing on the relationship between genetic risk scores for BMI, inflammation and MDD	406 participants aged 48.7 years on average with an s.d. of 15.1 years, 45.3% were male and 54.7% were female from a research project on mental and physical health in the general population of London, UK	The study found that higher BMI, influenced by genetic risk scores, was associated with increased levels of inflammatory markers like IL-6 and CRP, suggesting that BMI is a significant mediating factor in inflammation observed in MDD	The cross-sectional study limits the ability to capture establish causality and variables such as BMI might confound the effects, complicating the interpretation of how inflammation levels influence MDD
Zainal and Newman^70^	Observational study based on two waves of blood sample collection approximately 9 years apart, to identify markers of inflammatory activity (IL-6, CRP and fibrinogen) measured using the ELISA technique	A sample of 945 participants (52.78% women) with an average age of 54.33 years	The study found that inflammation may be a risk factor for the development or relapse of MDD. Higher levels of inflammatory activity predict the development or relapse of MDD in individuals, particularly among women, younger adults, those with chronic health issues and low-income individuals	The reliance on self-reported data for both inflammatory markers and MDD diagnosis, which may introduce bias or inaccuracies. The study's sample size and demographic composition may limit the generalisability of the findings to broader populations
Green et al^71^	Longitudinal observational study analysing the association between systemic inflammation and symptoms of depression	880 participants 271 were cases of MDD and 609 were healthy controls without specific mention of gender or age distribution in the reviewed section. Data collected included information on serum CRP levels, CRP DNA methylation, depressive symptoms and structural characteristics of the brain via magnetic resonance imaging	The study found significant links between inflammation markers and brain structure in individuals with MDD. Higher serum CRP levels were correlated with reduced cortical thickness in key brain areas like the left superior frontal gyrus, left insula and left superior temporal gyrus. Additionally, levels of IL-6 and CRP were associated with specific symptoms, including changes in appetite and concentration	Lack of detailed description of methodology and control measures for confounding variables. The results may not be generalisable because of limitations of the study population
Czysz et al^72^	Observational research conducted to compare various inflammatory markers to assess their role as moderators of treatment outcomes in depression	The study evaluated 665 adult out-patients aged 18–75 years with chronic or recurrent non-psychotic MDD, divided into three groups: escitalopram plus placebo, escitalopram with bupropion SR, and venlafaxine SR with mirtazapine. It included a 3-month acute phase and a 4-month extension for non-responders, with biomarker blood samples collected from 166 consenting patients at baseline and week 12	The study analysed immunomarkers such as IL-13 to assess their influence on antidepressant treatment efficacy in depression. It aimed to determine how these inflammatory markers affect treatment outcomes. Findings indicated that higher IL-13 levels were associated with better remission rates in specific antidepressant combinations	The study faced limitations such as a small sample size and exclusion of some immunomarkers, limiting its scope. Unaccounted factors like lifestyle and medication use, along with potential measurement errors, could affect the findings, making the results less generalisable across different populations
Kofod et al^45^	Observational cross-sectional study	90 adult participants aged 18 years or older with a diagnosis of moderate severity depression	The study found correlations at baseline between inflammatory markers and symptom severity on specific dimensions of symptoms, but not with overall depression severity. The study did not find any association between inflammatory markers before or during the antidepressant trial and the differential risk of psychiatric hospital admissions over 10 years	Limitations of the study include sample size, lack of information on certain confounders and lack of control for multiple testing in the analyses
Comai et al^73^	Observational cross-sectional study on patients suffering from bipolar depression and major depression	Sample of 166 patients, including 100 with MDD and 66 with bipolar disorder, aged 18–65 years	The study revealed that patients with bipolar depression exhibit higher kynurenine levels and kynurenine/tryptophan ratios compared with those with unipolar depression. Additionally, lower tryptophan levels correlate with more severe depressive symptoms across diagnoses. Also, changes in white matter microstructure may serve as a potential biomarker for bipolar disorder	Small sample size. Selection of participants from a single centre. Uncontrolled variables: participants’ lifestyle and possible use of drugs not taken into consideration
Swoboda et al^74^	Observational pilot study	129 patients with a current major depressive episode, divided into 63 not on antidepressants and 66 on stable treatment for at least 2 weeks. Additionally, 61 healthy controls and 39 patients with MDD in remission were recruited. A follow-up included 59 untreated and 60 treated patients, all receiving standard treatment during the observation period	The study evaluated MIF levels in serum and peripheral blood mononuclear cells, finding no significant link between baseline MIF levels and depression severity 3 weeks after inclusion. However, higher MIF levels at inclusion were associated with a poorer prognosis in MDD. Patients experiencing a major depressive episode displayed moderate to high severity based on three assessment scales, but these scores improved following therapy. Additionally, elevated MIF expression at inclusion correlated with smaller improvements in depression scales (HRSD and MADRS), especially in non-medicated and female patients	Small sample size. Lack of consideration for confounding factors. Absence of a matched control group. Pilot study design may limit statistical power
Lan et al^75^	Observational cross-sectional analysis of patients with MDD, examining the association between BMI and inflammation	Sample of 265 patients with MDD, divided into groups based on weight (underweight, normal weight, overweight/obesity)	The study found that patients with MDD who were overweight or obese had higher plasma levels of TNF-α, IL-8 and MIP-1β, and exhibited poorer performance in processing speed and working memory. The results suggest that increased inflammation may be a biological mechanism connecting higher BMI with cognitive deficits in processing speed among patients with depression	Cross-sectional design limits the ability to infer causality between obesity, inflammation and cognitive dysfunction. The study population was limited to a specific group (MDD), which may not generalise to the broader population
Elgellaie et al^46^	Observational study on patients with MDD not in treatment versus non-depressed controls	60 individuals with MDD and 60 controls with an age between 18 and 54 years, with a mean of 25.05 years and s.d. of 6.61 years. The sample consisted of 34 women and 26 men in both study groups	Significant links were found between IL-1α and IL-6 levels and psychopathological symptoms, with distinct gender differences. Females with depression had significantly higher IL-6 levels than those in the control group, whereas no notable differences were found in IL-6 levels between males with depression and healthy males. Overall, patients with MDD showed higher concentrations of these markers compared with controls	Lack of a treated control group limits the study's ability to directly compare the effect of treatments on the presence of pro-inflammatory cytokines in patients with major depression. Limited sample size to ensure complete representation of the MDD population
Liu et al^76^	Observational cross-sectional study from longitudinal cohort	790 treatment-naïve individuals, aged between 34 and 84 years, who had not taken psychotherapeutic drugs and had no history of psychotherapy. The population was included both genders though specific age and gender distribution details are not provided in the snippet	The study found no significant correlation between levels of IL-6, IL-8 and other inflammatory markers with depression. Additionally, the use of antipsychotic and anxiolytic drugs did not significantly affect pro-inflammatory cytokine production in patients with psychological disorders. This suggests that in this participant cohort, there is no clear link between inflammation levels and depression, nor a significant influence of psychotropic medications on inflammation	The cross-sectional nature of the study limits the ability to draw causal relationships. The results may not be generalisable beyond the specific cohort studied. Interactions between depression, anxiety and stress, because these psychological conditions can interact synergistically to enhance broader inflammatory responses
Hu et al^47^	Observational network study to explore the relationship between depression and inflammation	Not specified	Study findings may include the identification of depression-related genetic targets, analysis of inflammatory pathways involved in the inflammatory response in the brain and the association of pro-inflammatory factors such as IL-6 and TNF-α with depression.	Intrinsic limitations of the observational design. Need to confirm the results through randomised controlled clinical studies
Xie et al^77^	Observational research that focuses on the analysis of inflammation in astrocytes in patients with MDD	A sample of 70 patients with MDD and 70 healthy controls was included in the study. The characteristics of the participants did not show significant differences in terms of gender, age and BMI between the two groups	The study focuses on inflammation in astrocytes in patients with MDD, analysing extracellular vesicles derived from astrocytes and detecting a significant increase in various inflammatory markers in the serum and extracellular vesicles of patients with MDD compared with healthy controls. The results support the hypothesis of glial inflammation in depression. This study is the first to examine a wide panel of inflammatory cytokines in patients with depression	The small size of astrocyte-derived extracellular vesicles may hinder the ability to perform comprehensive high-throughput omics assays like proteomics, transcriptomics and lipidomics, thus limiting a full understanding of the cytokine analysis involved in inflammation. Additionally, despite the kit's sensitivity to nine cytokines, the limited range analysed might not fully capture the complexity of the inflammatory profile associated with MDD
Hursitoglu et al^78^	Observational case–control study comparing treatment-naive, non-smoking, first-episode patients with MDD versus healthy controls matched for age, gender and BMI	100 individuals divided into 50 patients with MDD and 50 healthy controls. Ages ranged from 18 to 65 years, with an equal distribution of genders across both groups	Higher levels of serum NOX1 and Raftlin were found in patients with MDD compared with healthy controls, indicating their potential as diagnostic markers for MDD	Limited generalisability owing to the small and specific sample size, absence of long-term follow-up and the biomarkers were only measured in serum, not reflecting central nervous system processes
Bai et al^79^	Observational study cross-sectional	The study sample includes 133 patients with bipolar disorder and depressive disorder versus 86 healthy controls	The study found significantly higher levels of NfL and P1NP in patients with bipolar disorder and MDD compared with healthy controls. Additionally, a positive correlation between P1NP levels and TNF-α in the bipolar disorder group suggests an association between the brain–bone axis, systemic inflammation and cognitive function in these disorders	Sample size. Generalisability: the study focused on patients with severe affective disorders, it may be difficult to generalise the findings to other conditions or populations
Mandal et al^80^	Prospective, clinic-based cohort study	81 patients aged 18–65 years diagnosed with MDD according to the ICD. Of these, 31 completed the study allowing for analysis of inflammation marker levels across several weeks	The study measured changes in pro-inflammatory (TNF-α) and anti-inflammatory (IL-10) markers over an 8-week period. It found that TNF-α levels increased, whereas IL-10 levels decreased from baseline to 8 weeks, although these changes were not statistically significant	High attrition rates and a small sample size limited data completeness and generalisability. The 8-week study duration may have been too short to observe significant changes in inflammatory markers
Sánchez-Carro et al^81^	Observational longitudinal cohort study	Sample of 171 patients, including 91 with depression (71.42% women, mean age = 50.64 years) and 80 healthy controls (67.50% women, mean age = 49.12 years)	The study used machine learning to differentiate between patients with MDD and healthy individuals by analysing immunometabolic markers and lifestyle factors. The results confirmed that these markers and factors effectively classify individuals with major depression, highlighting the potential of machine learning in diagnosing and understanding various forms of depression	Longitudinal design requires extensive resources and time, which might limit the feasibility of similar studies. Potential biases owing to self-reported data on depressive symptoms and lifestyle factors
Foley et al^82^	Observational longitudinal study	86 participants recruited through UK health services. Participants included individuals with depressive episodes who met ICD-10 criteria for depressive episodes	The study results highlighted the importance of IL-6 activity as a biomarker in depressions, with particular focus on clinical and cognitive effects. Significant associations were identified between IL-6 activity and various clinical and cognitive outcomes in study participants	The study, being observational, did not manipulate experimental variables, limiting definitive conclusions about IL-6's causal effects on inflammation and cognitive functions in depression. Additionally, the small sample size of 86 participants may restrict the generalisability of the findings
Moilanen et al^83^	Observational cross-sectional study	5443 middle-aged participants from a large sample of the Finnish general population. The BDI-II was used to screen depression	The results reinforce the link between depression and inflammation, showing that depressive symptoms correlate with increased levels of hsCRP. Specifically, participants with atypical depressive symptoms were 2.59 times more likely to have elevated hsCRP levels than non-depressed individuals. Those with non-atypical depressive symptoms also showed a connection to inflammation, albeit weaker and still statistically significant	The findings from a middle-aged Finnish population may not apply broadly to other populations or ethnic groups, given potential genetic, environmental or lifestyle variations. The use of a self-administered BDI-II questionnaire for diagnosing depression might lack the accuracy of a detailed clinical evaluation. Furthermore, categorising depressive symptoms as atypical or non-atypical might oversimplify the complexity of these symptoms and their association with inflammation
Sarmin et al^84^	Observational case–control study	217 (110 patients with MDD, 107 healthy controls), adults, matched by age, gender and BMI. Teaching hospital and general community in Dhaka, Bangladesh	Significant elevation in serum levels of IL-12 and IL-4 in patients with MDD compared with healthy controls, with positive correlation between the levels of these cytokines and the severity of depression	The study is limited as it only tracks two cytokines and doesn't reflect the full pathophysiology of MDD or consider influences like diet and physical activity. Its findings, focused on the Bangladeshi population, may not apply to other groups because of cultural and genetic differences
Zeng et al^48^	Observational study	The study involved 281 individuals diagnosed with MDD from psychiatric clinics, consisting of 125 males and 156 females. The average age of male patients with suicidal ideation was 31.4 years (s.d. 11.2), compared with 35.2 years (s.d. 12.6) for those without. Female patients with suicidal ideation averaged 32.1 years (s.d. 11.3), whereas those without averaged 34.6 years (s.d. 11.9). Regarding education, male patients with suicidal ideation had an average of 12.2 years of education (s.d. 3.1), similar to males without ideation at 12.1 years (s.d. 3.1). Female patients with ideation had slightly more education, averaging 13.1 years (s.d. 3.2), compared with 12.2 years (s.d. 3.4) for those without	The analysis revealed a statistically significant indirect effect of sleep disruption on suicidal ideation through inflammatory markers such as IL-6 and TNF-α. Additionally, the study identified differences in plasma cytokine levels between MDD patients with and without suicidal ideation using statistical analyses	The study may have been limited by the sample size and the specific criteria used for participant selection, potentially affecting the overall representation of individuals with MDD and suicidal ideation. Furthermore, the reliance on self-reported measures for assessing sleep disruption and suicidal ideation may introduce bias and affect the accuracy of the results

MDD, major depressive disorder; CRP, C-reactive protein; TNF, tumor necrosis factor; IL, interleukin; CSF, cerebrospinal fluid; CXCL, C-X-C motif chemokine ligand; sTNFR, soluble tumour necrosis factor receptor; BDI-FS, Beck Depression Inventory-Fast Screen; MARS, medication adherence rating scale; STAR*D, sequenced treatment alternatives to relieve depression; AUC, area under the curve; CCL, C-C motif chemokine ligand; HRSD, Hamilton Rating Scale for Depression; BMI, body mass index; ELISA, enzyme-linked immunosorbent assay; MIF, Macrophage Migration Inhibitory Factor; MADRS, Montgomery–Åsberg depression rating scale; MIP, macrophage inflammatory protein; NOX1, NADPH oxidase 1; NfL, neurofilament light chain; P1NP, N-terminal propeptide of type 1 procollagen; BDI-II, Beck Depression Inventory-II; hsCRP, high-sensitivity C-reactive protein.

**Table 4:** Randomised controlled trials

Author	Type of study	Analysed sample	Results	Limitations
Liu et al^76^	Evidence-based multicentre randomised controlled study that utilises serological analysis to examine patients with depression	158 participants with an average age of 27.5 years, of which 62.6% were women, divided into subtypes of major depression: melancholic, atypical and anxious	Significant differences were observed in the levels of IL-6 and TNF-α among patients with different remission states and among patients with melancholic, atypical and anxious depression	Sample size. Duration of follow-up. Generalisability of the results: since the study may have involved a specific population of participants with certain demographic or clinical characteristics
Liu et al^85^	Randomised controlled trial investigating the dose–response effects of mindfulness-based cognitive therapy on undergraduate students with MDD	60 undergraduate students diagnosed with MDD excluding those with other significant mental or physical health issues. Specific details on gender distribution and exact age range are not mentioned	The study found that participants in the mindfulness-based cognitive therapy group experienced significant improvements in depression, anxiety and sleep quality. Additionally, they showed decreased levels of inflammatory markers (IL-1β, IL-6, IL-8, TNF-α) and increased levels of BDNF, compared with those in a wait-list control group	The study's limitations are its small sample size and brief duration, affecting the findings’ generalisability and sustainability, particularly given the specific population of undergraduate students

IL, interleukin; TNF, tumor necrosis factor; MDD, major depressive disorder; BDNF, brain-derived neurotrophic factor.

### IL-1β

IL-1β plays a significant role in depression. It has been detected in various tissues, including blood, cerebrospinal fluid, and brain tissues, and elevated levels have been observed in patients with depression. The increase in IL-1β can affect neurotransmitters and contribute to the activity of other neuroinflammatory pathways, thereby contributing to the pathogenesis of depression.^43,46,52,58,61,85^

### IL-6

IL-6 was identified as a strong indicator of the severity of depressive symptoms compared with healthy controls, with a significant correlation to anhedonia and reduced motivation. Both longitudinal and cross-sectional studies have demonstrated that high concentrations of IL-6 are associated with an increased likelihood of depressive symptoms, particularly in patients with a high body mass index.^50,69,82^ Further studies have extended this correlation to CRP levels, linking IL-6 and CRP to depressive symptoms such as sadness, loss of interest and difficulty concentrating.^65^

### CRP

Various research has shown that high levels of CRP are consistently linked to an increased risk of depressive symptoms, particularly affecting appetite and concentration. The analysis of large samples indicates that CRP is a strong predictor of depression, emphasising the central role of systemic inflammation in the pathogenesis of depression.^67,68,83^ Other studies have shown that high plasma CRP levels and increased TNF and soluble IL-6 receptors in the CSF are associated with severe depressive symptoms such as anhedonia and reduced motivation.^52,53,60,68,83^

### TNF-α

Some studies have highlighted that elevated levels of TNF-α, IL-8 and macrophage inflammatory protein 1β (MIP-1β) in plasma are correlated with worse performance on processing speed and working memory tests in patients with MDD and obesity.^75^ The interaction between TNF-α and fatigue in patients with depression suggests a direct influence on physical symptoms of depression.^62,64,80^ However, another study, despite identifying significant associations between levels of CRP and IL-6 and depressive symptoms, noted no such association for TNF-α, suggesting a selective relevance of certain cytokines in depression.^51^

### Imaging studies and neuroinflammation

It has been documented that peripheral inflammation associated with high levels of CRP affects specific brain regions critical for managing depressive states, with elevated serum levels associated with thinner cortical regions such as the left superior frontal gyrus and left insula.^71^ These observations are corroborated by a review, highlighting how pro-inflammatory markers can cause demyelinating effects on white matter, compromising its integrity and representing a pro-inflammatory condition associated with a severe depressive course resistant to treatment.^55^ At the same time, another study has identified inflammation as a risk factor for the development or relapse of MDD, with a significant impact, particularly in demographic groups such as women over 50 years of age.^70^ These studies collectively demonstrate an important connection between inflammation, biochemical dysfunctions and damage to white matter in various forms of depression, opening new perspectives for research.

### Other pro-inflammatory markers in mood disorders

Elevated levels of pro-inflammatory cytokines such as IL-17, IL-21 and IL-23, and reduced levels of IL-35, have been frequently observed in patients with MDD compared with healthy controls, suggesting an enhanced pro-inflammatory gene expression.^63^ Emphasising the role of cytokines and the immune response in the pathogenesis of depression, another study demonstrated a significant increase in serum levels of IL-12 and IL-4 in patients with MDD, with a positive correlation between these cytokine levels and the severity of depression.^84^

### Differences in biomarkers between bipolar disorder and MDD

Monitoring of inflammatory biomarkers has played a crucial role in distinguishing between bipolar disorder and MDD, identifying significant differences in biomarker levels between these conditions.^66,72^ Markers such as IL-9, TNF-α, C-C motif chemokine ligand (CCL)3, CCL4, CCL5, CCL11, CCL25, CCL27, C-X-C motif chemokine ligand (CXCL)6 and CXCL11 have been indicative of bipolar disorder, whereas IL-1β, IL-2, IL-4, IL-6, IL-7, IL-10, IL-16, CCL20, CCL21, CXCL12, platelet-derived growth factor subunit B and vascular endothelial growth factor are more frequently associated with MDD. Research a year earlier on depression and bipolar disorder also found higher levels of IL-1β, TNF-α, soluble tumour necrosis factor receptor (sTNFR)1, IL-12 and IL-10 in patients with MDD, compared with higher levels of IL-6, sTNFR2, IL-18, IL-33, soluble suppression of tumourigenicity 2 and neuroprotective gene modulator KLOTHO in patients with bipolar disorder.^61^

### Role of macrophages and oxidative stress

The macrophage migration inhibitory factor (MIF) and oxidative stress have been explored as potential markers of antidepressant treatment efficacy, indicating that these factors may play a role in the responses to antidepressant treatment.^74^ Concurrently, another study investigated the role of oxidative stress by examining the serum levels of two potential biomarkers that might be involved in the pathophysiology of MDD: NOX1, also known as NADPH oxidase 1, an enzyme involved in various physiological and pathological processes, including oxidative stress and inflammation; and Raftlin, a protein important in cellular processes, including those related to oxidative stress and inflammation.^78^

### Tryptophan metabolism and neuroinflammation

Studies on how alterations in tryptophan metabolism affect neuroinflammation have confirmed a reduction in serotonin levels in favour of kynurenine, which exacerbates depressive symptoms.^62,73^ These metabolic changes may also correlate with structural damage to white matter, suggesting potential treatment resistance in cases of severe depression.

### Cognitive impact of inflammation

It has been demonstrated how inflammation affects cognitive function and brain structure, through biomarkers such as neurofilament light chain (NfL) and the N-terminal propeptide of type 1 procollagen (P1NP), which show a correlation with the severity of symptoms and cognitive decline in patients with mood disorders.^79^

### Therapeutic implications and treatment responses

Cognitive–behavioural therapy-based interventions have been shown to reduce symptoms of depression, anxiety and sleep difficulties, consequently lowering inflammatory markers such as IL-1β, IL-6, IL-8, TNF-α and increasing levels of BDN.^85^ A similar conclusion has been reached by other studies, which have highlighted the use of psychotherapies, anti-inflammatory drugs and nutritional strategies to reduce inflammation for the improvement of depressive symptoms.^54,56^ A direct link between inflammation and the efficacy of depression treatment is also suggested by improved responses to antidepressant treatments associated with high levels of IL-13.^72^ Recently, the hypothesis of the pathogenic role of inflammation in depression has been strengthened, indicating that high cytokine levels are linked to the severity of the disorder, particularly prevalent in treatment-resistant depression. These findings promote the use of anti-inflammatory therapies as promising strategies to manage cases of treatment-resistant depression.^58^

### New research perspectives

The use of extracellular vesicles derived from astrocytes has provided new insights into glial inflammation in depression, suggesting that astrocytes can be a useful tool for analysing the state of astrocytes in human patients.^58^

### Application of artificial intelligence in depression classification through inflammatory biomarkers

Machine learning techniques have been utilised to distinguish between patients with depression and healthy individuals, highlighting the effectiveness of immunometabolic markers and lifestyle-related factors in classifying various forms of depression. These findings demonstrate how these tools can offer new perspectives in the recognition and management of depression, and underscore the central role of cytokines and the immune response in the pathogenesis of the disorder.^77,81^ These data illustrate the complexity and specificity of the immuno-inflammatory mechanisms in depression, and underscore the importance of more targeted and sophisticated diagnostic and therapeutic approaches.

However, the studies under review also reveal several significant limitations that could influence the interpretation of the results.

### Variability in measurement method

The techniques used to measure inflammatory biomarkers vary significantly between studies, potentially affecting the comparability of results. Some studies use peripheral blood samples, whereas others examine markers in CSF or through brain imaging, which may not reliably reflect neuroinflammatory conditions.^52,53,68^

### Limits of correlation between peripheral and central cytokines

Recent research has indicated that the correlation between cytokine levels in the blood and CSF is less direct than previously assumed.^57^ It has been reported that, despite correlations in specific subgroups, peripheral biomarkers generally do not reliably match those in the CSF, limiting their use as direct indicators of brain inflammation. This challenges the use of peripheral cytokines as accurate reflections of neuroinflammatory conditions, highlighting the need for further research to clarify these dynamics and improve diagnostic methodologies.

### Study design and causality

Many of the studies included in this scoping review are cross-sectional observational studies, which limits the ability to establish causal relationships between levels of inflammatory biomarkers and depressive symptoms. For example, it is unclear whether inflammation causes depression or *vice versa*.^44–46,59–63,73,75^

### Generalisability of results

Studies focus on specific populations or demographic groups, which limits the generalisability of the results to broader or different contexts than those studied. For example, some findings may not be applicable to populations with different ethnic backgrounds or health conditions.^59,60,63,68,70^

### Overlap between conditions

Many patients with major depression may also suffer from other medical conditions that could influence levels of inflammatory biomarkers, introducing potential confounders into the study results.^63,68,69^

### Timing and dynamics of inflammation

The temporal dynamics of inflammation in relation to the onset and progression of depressive symptoms are not yet fully understood.^70,75,82^ This uncertainty necessitates further longitudinal studies to determine whether inflammation precedes, coincides with or follows the development of depressive symptoms. Incorporating these limitations into the results helps to better contextualise the findings and suggests areas for future research, which are crucial for refining our understanding of the link between inflammation and depression, and for developing more effective and targeted treatment strategies.

## Discussion

Previous studies have established an almost constant link between depression and the presence of inflammatory elements in both the innate and adaptive immune systems. Our understanding of the role of inflammatory biomarkers in MDD has significantly evolved, revealing a complex connection between systemic inflammation and the clinical manifestations of depression.^55^ This hypothesis suggests that inflammatory processes may trigger, exacerbate or sustain depression; however, the temporal sequence of inflammation relative to the onset and progression of depressive symptoms remains uncertain.^82^ In particular, intensified cytokine responses to pathogens and stress have been observed in patients with depression, which, when combined with other predisposing factors, can lead to prolonged inflammation and the dysregulation of various physiological axes.^60,75^ Although inflammatory cytokines have been extensively studied, the correlation between specific biomarkers and clinical symptoms remains complex and influenced by biological, psychological and environmental factors.^61,63,66,72,84^ Our scoping review has identified numerous studies that highlight a growing consensus on the role of IL 1β, IL-6, TNF-α and CRP in modulating the severity and treatment response of depression.^52,60,65^ The latest research emphasises the role of the cytokines IL-6 and TNF-α, correlated with greater severity of depressive symptoms, including anhedonia and reduced motivation, particularly when CRP levels are elevated.^46–48^ Furthermore, IL-6 not only signals the presence of depression, but can also indicate resistance to conventional antidepressant treatments, suggesting the need for personalised therapeutic strategies.^69,82^ These findings broaden our understanding of the role of inflammation in depression and may guide the development of more targeted and effective interventions. Imaging studies have revealed that chronic inflammation can impair neuroplasticity, reducing the brain's ability to form new synaptic connections and restructure existing ones. This deterioration is particularly pronounced in brain areas critical for mood and cognitive regulation, such as the left superior frontal lobe, left insula and left superior temporal lobe. A direct correlation has been observed between high levels of serum CRP and cortical thinning in these regions.^50,56,60,62,64,70,73,75,80,85^ Concurrently, alterations in tryptophan metabolism that increase the production of kynurenine at the expense of serotonin further exacerbate depressive symptoms. These biochemical changes illustrate a mechanism through which inflammation can worsen depression, creating a vicious cycle of inflammatory response and neuropsychological deterioration.^62,73^ The hypothalamic-pituitary-adrenal axis, sensitive to the effects of inflammation, can also influence the development and course of depression, confirming a further connection between inflammatory responses and psychiatric dysfunctions.^42^ The use of large databases such as the UK Biobank has allowed for the examination of the impact of inflammation on a large scale, revealing how age can affect the inflammatory response and the severity of depressive symptoms, especially in individuals over 50 years old. These individuals also tend to show a stronger correlation between the levels of cytokines such as IL-6 and CRP and the severity of depressive symptoms.^50^ This phenomenon, often attributed to an increase in systemic inflammation owing to ageing, known as ‘inflammaging’, is enhanced by immune changes that accumulate with chronic stress over the lifespan. It has been confirmed that inflammation may represent a potential risk factor for the development or relapse of MDD, highlighting the need for personalised treatment strategies that consider age and other demographic factors.^70^ This reinforces the importance of further longitudinal and interventional studies that include age as a key variable. Another critical aspect is the treatment and management of depression based on biomarker levels. The use of anti-inflammatory pharmacotherapy has been proposed, particularly in cases resistant to conventional treatment,^58^ whereas other research groups^56^ highlight the importance of integrating anti-inflammatory and nutritional approaches in the treatment of depression. Recently, it has been shown how mindfulness-based cognitive therapy can reduce symptoms of depression, anxiety and sleep disturbances, linking these improvements to a decrease in inflammatory markers such as IL-1β, IL-6, IL-8, TNF-α and increased BDNF.^85^ These results suggest a link between psychotherapeutic interventions and biological changes, opening prospects for reverse treatments, such as anti-inflammatory ones, to improve symptoms. Some researchers, also explored how antidepressants affect the inflammatory response.^74^ The integration of anti-inflammatory treatments and psychotherapeutic interventions has shown potential in reducing depressive symptoms and lowering levels of inflammatory cytokines in the blood, demonstrating that it is possible to positively influence both the psychological and biological aspects of the disease. Although the potential to personalise treatment based on biomarker levels is promising, individual variability in response to antidepressant treatments and the complexity of the inflammatory pathways involved make this a complex area that requires further research. Longitudinal studies and RCTs will be crucial to determine whether modifications to inflammatory pathways can indeed improve clinical and therapeutic outcomes in these patients. Despite these significant prospects, it is important to highlight the considerable methodological limitations that might affect the interpretation of the results. Most studies on biomarkers in depression rely on cross-sectional and observational designs, which, although useful for identifying associations, are incapable of establishing causality. This is particularly relevant because cross-sectional designs cannot discern whether inflammation is a cause or a consequence of depression. The generalisability of the results is limited because the studies focus on specific populations or demographic groups. This implies that the results may not be applicable to broader or different contexts than those studied, such as populations with different ethnic backgrounds or health conditions.^59,60,63,68,70^ Variation in detection techniques and differences in the cohorts studied further reduce the ability to generalise the results. For example, the protocols for measuring biomarkers vary widely: some studies measure these markers in peripheral blood samples, whereas others analyse them in CSF or investigate them through brain imaging techniques. Such methodological differences can generate inconsistent and sometimes contradictory results, making comparisons and data synthesis complex.^52,53,55,68,71,74^ However, the main concern remains the validity of measuring brain inflammation through peripheral biomarkers. A recent meta-analysis has raised important questions about the accuracy with which cytokine levels in peripheral blood can represent inflammation in the CSF.^57^ The conclusions suggest that, although some correlations may be found in specific patient subgroups, generally, peripheral inflammatory biomarkers do not reliably correspond to those in the CSF, thus questioning the use of such measurements as direct indicators of cerebral inflammation. This implies a need for greater caution in interpreting these markers as diagnostic tools in depression and calls for further research to develop more precise and representative measurement methodologies for inflammation at the level of the central nervous system. The necessity for extensive and standardised studies is indispensable to validate the diagnostic and therapeutic utility of inflammatory biomarkers.

Concluding the discussion, the complexity of the relationship between inflammation and MDD is confirmed, suggesting greater caution in the use of peripheral inflammatory biomarkers as indicators of neuroinflammation. It underscores the need for careful interpretation of peripheral cytokine measurements, where methodological variability and confounding factors may influence the results, thereby complicating the establishment of direct correlations between peripheral and central brain cytokine levels. Further research is essential to enhance measurement techniques for biological mechanisms and to integrate these findings with psychosocial and environmental factors. Together, these elements significantly contribute to the complexity of MDD and its therapeutic management.

This review highlighted the growing interest in inflammatory biomarkers in depression, as demonstrated by the increasing number of studies and scientific research published in recent years. However, the scientific literature on this topic exhibits several biases. First, the geographic origin of most studies is predominantly limited to Europe, Asia and North America. Second, there is a lack of consistency in the methods employed and the conclusions drawn, making it challenging to compare results and reach definitive conclusions. Third, the correlation between cytokine levels in blood and cerebrospinal fluid is less straightforward than previously thought, and there is significant overlap between medical conditions that could influence levels of inflammatory biomarkers. The reviewed studies demonstrate that inflammation is not merely a peripheral phenomenon but profoundly influences the neurological and psychological processes underlying depression.

### Complexity of inflammatory biomarkers

Our study reveals a complex link between inflammation and MDD, highlighting both the challenges and opportunities associated with using inflammatory biomarkers for the diagnosis and potential treatment of this disorder. Inflammatory biomarkers, such as IL-1β, IL-6, TNF-α and CRP, have emerged as significant indicators of systemic inflammation associated with MDD. Along with other markers, they reveal their multifaceted nature and the complexity of interactions within the nervous system, underscoring the intricate relationship between inflammatory processes and depressive pathology. However, the correlation between the levels of these biomarkers in peripheral blood and CSF remains uncertain. This highlights the need to advance the precision of measurement methodologies to ensure that the assessment of cerebral inflammation is accurate and reliable.

### Future research directions

It is essential to conduct longitudinal and interventional studies with a broader range of markers and patient populations to validate the role of inflammatory biomarkers in MDD. Incorporating machine learning can help analyze large datasets, identify new biomarkers undetectable by traditional methods, and recognize early signs of depression before clinical symptoms appear. These studies should aim to clarify the causal links between inflammation and depression and optimize anti-inflammatory treatments for the disorder.

### Personalisation of treatment

The heterogeneity of inflammatory responses among patients with MDD underscores the need for personalised treatment strategies. Integrating therapeutic approaches that specifically target inflammatory pathways could significantly improve treatment efficacy, especially for those patients with forms resistant to conventional treatments.

### Implications for clinical practice

Clinicians should consider inflammation as a significant factor in the management of MDD, utilising information on inflammatory biomarkers to guide treatment decisions. Adopting a model of personalised medicine could not only enhance clinical outcomes, but also offer new insights into the understanding and treatment of depression.

Biomarker research in MDD faces numerous challenges, including an insufficient understanding of the aetiopathogenesis of the disorder, its notable heterogeneity, frequent comorbidities and the variable specificity of biomarkers. Additionally, the current methods and detection techniques present significant obstacles. The variability in biomarker measurements and the difficulty in correlating peripheral with central inflammation indicate an urgent need to refine these methodologies. Developing panels of advanced biomarkers and evaluating them through the use of new technologies could offer effective solutions to overcome these barriers, thus improving the accuracy and reliability of diagnoses and treatments in MDD.

## Data Availability

The data supporting the findings of this scoping review are available upon reasonable request from the corresponding author, W.P.
